# Maintaining phagosome integrity during fungal infection: do or die?

**DOI:** 10.15698/mic2020.12.738

**Published:** 2020-12-03

**Authors:** Mabel Yang, Glenn F.W. Walpole, Johannes Westman

**Affiliations:** 1 Program in Cell Biology, The Hospital for Sick Children, Toronto, ON, M5G 0A4, Canada.; 2 Department of Biochemistry, University of Toronto, Toronto, ON, M5S 1A8, Canada.

**Keywords:** Candida albicans, phagosome, lysosome, macrophage, fungi, calcium

## Abstract

Professional phagocytes represent a critical node in innate immunity and tissue homeostasis through their specialized ability to eat, drink, and digest material from the extracellular milieu. The degradative and microbicidal functions of phagocytes rely on the fusion of lysosomes with endosomal compartments such as phagosomes, resulting in the digestion and recycling of internalized prey and debris. Despite these efforts, several particularly dangerous infections result from a class of tenacious pathogens that resist digestion, often surviving and even proliferating within the confines of the phagosomal membrane. One such example, *Candida albicans,* is a commensal polymorphic fungus that colonizes ~50% of the population and can cause life-threatening infections in immunocompromised patients. Not only can *C. albicans* survive within phagosomes, but its ingestion by macropahges triggers a yeast-to-hyphal transition promoting rapid intraphagosomal growth (several microns per hour) while imposing a substantial mechanical burden on the phagosomal membrane surrounding the fungus. Preservation of membrane integrity is essential to maintain the hostile internal environment of the phagosome, a functionality of degradative enzymes and oxidative stress. Yet, biological membranes such as phagosomes have a limited capacity to stretch. Using *C. albicans* as a model intracellular pathogen, our recent work reveals a mechanism by which phagosomes respond to intraphagosomal growth of pathogens by expanding their surface area, and as a result, maintain the integrity of the phagosomal membrane. We hypothesized that this expansion would be facilitated by the delivery and fusion of membrane from extraneous sources with the phagosome. Consistently, macrophages respond to the yeast-to-hyphal transition through a stretch-induced release of phagosomal calcium, leading to recruitment and insertion of lysosomes that accommodate the expansion of the phagolysosome and preserve its integrity. Below, we discuss this calcium-dependent mechanism of lysosome insertion as a means of avoiding phagosomal rupture. Further, we examine the implications of membrane integrity on the delicate balance between the host and pathogen by focusing on fungal stress responses, nutrient acquisition, inflammasome activation, and cell death.

Insertion of lysosomes is required for canonical phagosome maturation. However, this maturation process is self-limiting; macrophages detect when adequate lysosomes have fused with the maturing phagosome and terminate further insertion. After engulfment, the yeast form of *C. albicans* can survive inside phagosomes, undergoing continuous growth, and more importantly, triggering a secondary wave of lysosome fusion (**Figure 1A**). This second wave of lysosome recruitment and insertion requires intact microtubules as well as the centripetal motor protein dynein. But what mediates the second wave of lysosome fusion? At a first glance, transient increases in calcium which mediate the activation of calcium-dependent SNARE proteins, were of obvious interest. Cytosolic calcium transients can be buffered with a membrane-permeable acetoxymethyl precursor of BAPTA (BAPTA-AM): a 'fast' cytosolic calcium chelator. In our study, BAPTA-AM was added 30 minutes post-infection - a time when canonical phagosome maturation had already been completed. Buffering cytosolic calcium transients impaired lysosome insertion, but unexpectedly, neither calcium from the endoplasmic reticulum nor extracellular calcium was required for this process. To understand the source of the calcium, changes in cytosolic calcium were monitored by expressing the genetically-encoded fluorescent calcium indicator GCaMP6s. Micro-fissures induced in the phagosomal membrane using LLOMe —a lysomotropic compound that accumulates in acidic compartments where it is converted into a lytic form—revealed that the lumen of the phagosome itself is a significant source of calcium. In turn, phagosome expansion requires the release of this phagosomal calcium pool triggering a secondary wave of lysosome fusion (**Figure 1A**). Previous work studying canonical phagosome maturation identified an essential role for the lysosomal transient receptor potential family channel-1 (TRPML-1) in local calcium release and phagosome maturation. Although local calcium release mediates the ongoing recruitment of lysosomes, phagosome expansion persisted in macrophages lacking TRPML-1. Additionally, the endolysosomal lipid PtdIns (3,5)P_2_, which activates TRPML-1, was also not required for phagosome expansion. As such, the second wave of lysosome fusion appears to bypass several hallmarks of canonical phagosome maturation. These findings may point towards an interesting case of convergent molecular evolution, whereby the second wave of lysosome fusion occurs by re-harnessing pre-existing calcium-dependent fusion machinery. Impaired lysosome fusion in macrophages treated with calmidazolium, a calmodulin inhibitor, implicates the involvement of SNARE proteins and tethering factors in the phagosome-lysosome insertion pathway. Thus, the cooperative action of previously implicated endolysosomal SNAREs, Rab GTPases, and tethering factors may help facilitate phagosome expansion and shed light upon the interplay of various membrane fusion machineries.

**Figure 1:**
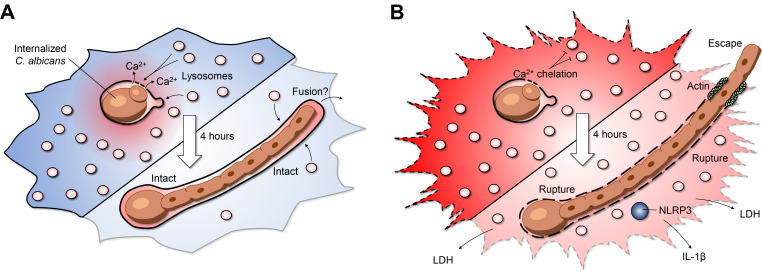
FIGURE 1: Lysosome Fusion Maintains Phagosome Integrity during Fungal Infection. **(A)** Phagosomes expand their surface area by insertion of lysosomes. As *C. albicans* initiates filamentation inside the mature phagosome, luminal Ca^2+^ is released, leading to recruitment and insertion of lysosomes. This preserves phagosome integrity while restricting fungal growth. **(B)** The second wave of lysosome insertion can be inhibited by applying the Ca^2+^ chelator BAPTA. Preventing cytosolic Ca^2+^ transients during intraphagosomal growth leads to early phagosome rupture, NLRP3 inflammasome activation, and subsequent macrophage cell death. Actin polymerizes at the site where the hypha protrudes through the plasma membrane, possibly re-sealing the macrophage membrane and preventing leakage of cytosolic content.

Compromising the host macrophages' ability to maintain phagosome membrane integrity can lead to dire consequences for the host cell. During the intraphagosomal growth of *C. albicans*, inhibition of lysosome insertion by calcium buffering triggered early phagosome rupture, detected by phagosomal leakage of a small fluorescent dye Sulforhodamine B. The resulting phagosomal rupture correlated tightly with host cell death, increased fungal growth, and escape of the hyphal tip from the host macrophage (**Figure 1B**). Accordingly, maintaining phagosome integrity is essential, not only for macrophage viability, but also to restrict the engulfed prey's growth. However, in the case of candidiasis, it remains to be explored if phagosome expansion ultimately improves fungal survival and persistence by 'cloaking' the organism from nearby immune cells. On the other hand, enhanced fungal growth could promote rupture and leakage of phagosome contents alerting nearby sentinel cells and engaging additional arms of the immune system to limit the infection. Consistent with the latter, our work identified that a major consequence of phagosome rupture is the activation of the NLRP3 inflammasome (**Figure 1B**). This multi-component protein complex can sense minute perturbations to the host cytosol and trigger the processing and secretion of the pro-inflammatory cytokines IL-1β and IL-18. Although inflammasome activation results in death of the infected macrophage, the process is altruistic in that these pro-inflammatory cytokines stimulate the recruitment of circulating neutrophils that may better limit fungal spread. Together, these observations raise the question of whether the fungus or the macrophage benefits from maintaining phagosome integrity as NLRP3 inflammasome activation could lead to different outcomes depending on the clinical setting. In the context of our study, it is clear that the fungus benefits from phagosome rupture despite NLRP3 inflammasome activation within infected macrophages.

Our work revealed that macrophages can accommodate the unrelenting intracellular growth of *C. albicans* infection by incorporating additional membrane derived from lysosomes. Beyond fungal infection, the data implicates lysosome insertion as a general response to intraphagosomal growth of pathogens and appears to have biological importance for other intracellular pathogens. For example, intraphagosomal growth of various fungi, bacteria, and protozoa have been described in macrophages. Indeed, phagosomes containing *Candida glabrata* and *Staphylococcus aureus* expand by lysosome insertion, albeit at a reduced rate, correlating with the reduced intraphagosomal growth of these prey. However, the factors that mediate the pre-initiation of lysosome-phagosome traffic and fusion in mature phagolysosomes are not apparent and could differ based on the identity of the intracellular pathogen. Macrophages containing filamentous fungi or bacteria may trigger a fissure-mediated fusion mechanism similar to *C. albicans*, but it is unclear if this is always the case. For example, *Salmonella enterica* remodels the host cell endolysosomal system by recruiting modified late endosomal compartments to expand the *Salmonella*-containing vacuole. In contrast, some pathogens may purposefully lyse the phagosome to access the nutrient-rich cytosol. Pathogens that manipulate phagosome maturation to prevent lysosome insertion clearly obtain a drastically different intracellular niche than that of *C. albicans*. Further investigation is required to uncover how the identified mechanism of phagosome expansion is modified by different intracellular prey.

A final intriguing finding was that disruption of phagosome integrity led to enhanced pathogen growth and escape (**Figure 1B**). Within the hostile environment of the phagosome, *C. albicans* exhibits transcriptional responses associated with starvation and oxidative/nitrosative stress. *C. albicans* is metabolically flexible and is known to adapt to the nutrient-deprived phagosome. For example, gluconeogenesis via the glyoxylate cycle has previously been reported as a metabolic adaptation inside the macrophage phagosome. Whole-genome microarrays were performed on RNA isolated from *C. albicans* grown in intact phagosomes. We confirmed that the fungal stress responses were maintained even after several hours of intraphagosomal growth. In contrast, a significant withdrawal in transcriptional stress responses were observed when phagosome expansion was inhibited. The downregulation of *C. albicans* genes associated with oxidative and nitrosative stress and metabolism compared to intact phagosomes demonstrates reduced microbiostatic capacity in ruptured phagosomes. In addition to benefiting from elevated pH and disseminating antimicrobial effectors, these findings support the notion that the escaping fungi gain access to the nutrient-rich cytosol. Collectively, these factors lead to increased fungal growth and escape. This final aspect of our study could have important implications for the fungus' ability to spread throughout tissues and cause systemic dissemination *in vivo*. However, even when phagosomes expand without interruption, the fungus ultimately escapes from the macrophage. Intriguingly, we observed that macrophages were able to survive this fungal escape without loss of plasma membrane integrity. As the hypha protrudes out of the cell membrane, actin polymerization mediated the formation of a tight seal, preventing cytosol leakage and consequent host cell death. How macrophages cope with the protruding hypha through the plasma membrane remains to be elucidated. Further investigation into the benefits and drawbacks of phagosome rupture in the context of pathogen growth, inflammasome activation, and host cell death are important and exciting aspects awaiting host-pathogen biologists.

